# Emergency Meningococcal ACWY Vaccination Program for Teenagers to Control Group W Meningococcal Disease, England, 2015–2016

**DOI:** 10.3201/eid2307.170236

**Published:** 2017-07

**Authors:** Helen Campbell, Michael Edelstein, Nick Andrews, Ray Borrow, Mary Ramsay, Shamez Ladhani

**Affiliations:** Public Health England, London, UK (H. Campbell, M. Edelstein, N. Andrews, M. Ramsay, S. Ladhani);; Public Health England, Manchester, UK (R. Borrow);; St. George’s University of London, London (S. Ladhani)

**Keywords:** meningococcal infections, meningococcal vaccines, meningitis/encephalitis, group W meningococcal disease, outbreak, Neisseria meningitides, bacteria, serogroup W-135, vaccination, meningococcal ACWY conjugate vaccine, MenACWY, epidemiology, teenagers, England

## Abstract

During the first 12 months of an emergency meningococcal ACWY vaccination program for teenagers in England, coverage among persons who left school in 2015, the first cohort to be vaccinated, was 36.6%. There were 69% fewer group W meningococcal cases than predicted by trend analysis and no cases in vaccinated teenagers.

Several countries in Europe, South America, and Australia are experiencing outbreaks of group W meningococcal (MenW) disease, caused by a hypervirulent strain of *Neisseria meningitides* belonging to sequence type 11 (ST11) clonal complex (CC) and associated with severe disease and a high case-fatality rate (*1*). In England, MenW cases caused predominantly by ST11 increased from 19 in epidemiologic year 2008–09 to 176 in 2014–15, which represented 2% and 24%, respectively, of all invasive meningococcal disease (IMD) cases (*2*).

In response to the national increase in MenW cases in England, an emergency immunization program with meningococcal ACWY conjugate vaccine (MenACWY) for adolescents was started in August 2015 (*3*). This program replaced the MenC program for children 13–14 years of age, and there was also a 2-year phased catch-up program for persons 14–18 years of age as of August 31, 2015 (Table 1) (*3*). Vaccine was also offered to new university entrants <25 years of age. Compared with persons of the same age who do not attend university, new university entrants have a higher risk for IMD, likely because of social factors that increase meningococcal transmission (*4*). This single-dose vaccination program aimed to directly protect vaccine-eligible cohorts and, in the long term, indirectly protect the wider population by reducing meningococcal carriage (*5*).

**Table 1:** Scheduling of meningococcal ACWY vaccination routines and 3-year catch-up vaccination programs, England*

Birth cohort	School year (age, y, at end of 2014–15 academic year on 2015 Aug 31)	Earliest school year at time of vaccination and type of vaccine received			
		2014–15	2015–16	2016–17	2017–18
2003 Sep 1–2004 Aug 31	6 (11)	NA	NA	NA	Y9 ACWY†
2002 Sep 1–2003 Aug 31	7 (12)	NA	NA	Y9 ACWY†	‡
2001 Sep 1–2002 Aug 31	8 (13)	NA	Y9 ACWY†	‡	‡
2000 Sep 1–2001 Aug 31	9 (14)	Y9 MenC§	NA	Y11 ACWY¶	‡
1999 Sep 1–2000 Aug 31	10 (15)	NA	Y11 ACWY¶	‡	‡
1998 Sep 1–1999 Aug 31	11 (16)	NA	NA	Y13 ACWY#	‡
1997 Sep 1–1998 Aug 31	12 (17)	NA	Y13 ACWY#	‡	‡
1996 Sep 1–1997 Aug 31	13 (18; those who left school in 2015)	Y13 ACWY#	‡	‡	‡

*NA, not applicable; Y, year. †New routine schedule MenACWY vaccination. ‡Completed MenACWY vaccination of cohort. §Routine schedule MenC vaccination. ¶School-based MenACWY catch-up cohorts. #General medical practice–based MenACWY catch-up cohorts.

Students in the target group who left secondary school in the summer of 2015 and were 18 years of age before September 2015 were the first cohort offered the vaccine through general medical practices, starting in August 2015. Nearly one third of this cohort were accepted into universities in 2015 (*6*). We report impact and vaccine effectiveness data for the first 12 months of the MenACWY program in England.

## The Study

Public Health England conducts enhanced national IMD surveillance in England, where 84% of the UK population resides. National Health Service hospital laboratories routinely submit local invasive meningococcal isolates to the Public Health England Meningococcal Reference Unit for confirmation and characterization, with national PCR testing also offered (*7*). Confirmed case-patients are routinely followed up for additional details, including vaccination history and outcome (*8*).

We assessed whether MenACWY vaccine coverage might be higher among new university entrants. We compared vaccine coverage in June 2016 estimated from data automatically extracted from primary care databases in university-affiliated (n = 79) medical practices on a university campus or recommended by the university on their website and non–university-affiliated (n = 7,543) general medical practices for persons who left school in 2015.

To estimate vaccine impact, we compared confirmed MenW, MenY, and MenB cases in persons who left school in 2015 with projected cases for the first academic year (September 2015–August 2016) after program introduction. To estimate projected case numbers for 2015–16 in the absence of vaccination, we fitted a Poisson regression model with age and time-trend parameters for case-patients 19–24 years of age during 2010–11 and 2015–16 who were not in vaccine-targeted cohorts. We used this model to estimate case projections and incidence rate ratios, which are presented as percentage decrease (1 – incidence rate ratio). MenC cases were excluded because of successful MenC vaccination programs for persons 14–16 years of age and new university entrants available since September 2013.

We assessed vaccine effectiveness among persons who left school during the 2015–16 academic year. Vaccine effectiveness was estimated by using the screening method (*9*). Vaccine coverage in cases was compared with population vaccination coverage in age-matched peers in England (*10*).

MenW cases in England increased overall by 15%, from 189 in the 2014–15 academic year to 218 in 2015–16. Isolates were available for 178 culture-confirmed cases in 2015–16; a total of 155 (87%) were ST11 CC. Case numbers and incidence increased in every age group except persons 15–19 years of age (26 to 18 cases; 31% reduction) and infants <1 year of age (26 to 17 cases; 35% reduction) (Table 2). Six (33%) of 18 teenage case-patients died, but no infant case-patients died.

**Table 2:** Age distribution of laboratory-confirmed cases of group W meningococcal infection in students, by academic year, England, 2010–11 to 2015–16*

Age group, y	Academic year											
	2010–11		2011–12		2012–13		2013–14		2014–15		2015–16	
	No. cases	Incidence	No. cases	Incidence	No. cases	Incidence	No. cases	Incidence	No. cases	Incidence	No. cases	Incidence
<1	3	0.45	4	0.59	3	0.43	12	1.77	26	3.91	17	2.56
1–4	8	0.31	6	0.23	5	0.19	5	0.18	22	0.80	26	0.94
5–14	1	0.02	1	0.02	3	0.05	1	0.02	4	0.06	7	0.11
15–19	4	0.12	1	0.03	13	0.40	13	0.40	26	0.80	18	0.56
20–24	3	0.09	1	0.03	3	0.08	7	0.19	6	0.17	18	0.50
25–44	1	0.01	3	0.02	6	0.04	6	0.04	7	0.05	10	0.07
45–64	7	0.05	6	0.04	13	0.10	12	0.09	29	0.21	43	0.31
>65	11	0.13	11	0.13	15	0.17	34	0.37	69	0.72	79	0.81
Total	38	0.07	33	0.06	61	0.11	90	0.17	189	0.35	218	0.40

*Academic years are September 1–August 31. Incidence, cases/100,000 population.

**Figure 1:**
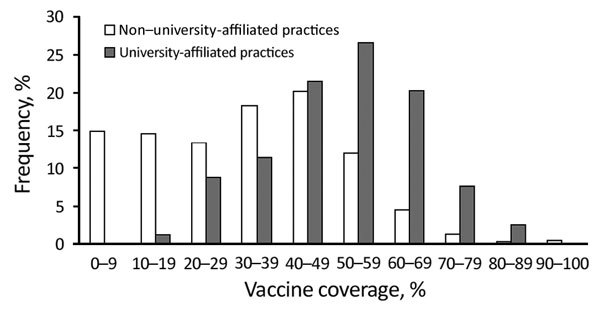
Frequency distribution of meningococcal ACWY conjugate vaccine coverage among teenagers who left school in 2015 in university-affiliated (n = 79) and non–university-affiliated (n = 7,543) general medical practices, England, June 2016. University-affiliated medical practices are either on campus or recommended by universities. The list might not be comprehensive, and non–university-affiliated medical practices will still register students.

By June 2016, vaccine coverage in persons who left school was 36.6%; a total of 79% of these vaccinations were administered during August–September 2015. Vaccine coverage among persons who left school was higher in university-affiliated medical practices than in non–university-affiliated medical practices (56.1% vs. 33.8%; p<0.0001) (Figure 1).

**Figure 2:**
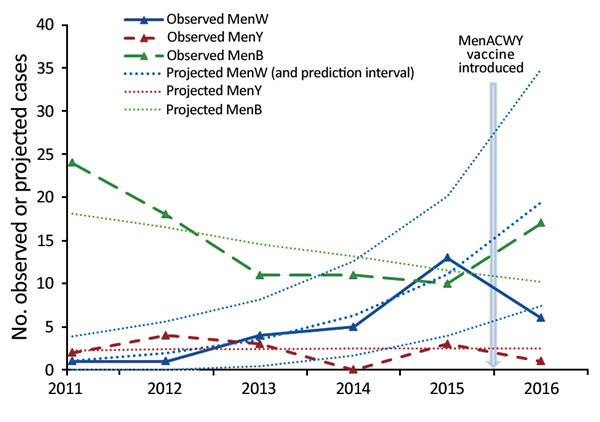
Observed and projected cases of W, Y, and B invasive meningococcal disease in England determined on the basis of trend lines fitted to the prevaccination period (November 2010–11 to 2014–15) and extrapolated to the 2015–16 academic year for the cohort with group W, Y, and B invasive meningococcal disease and who left school. Men, meningococcal.

During the first 12 months of the MenACWY vaccination program for teenagers, there were 6 confirmed MenW cases among ≈650,000 persons who left school compared with a projected 19.4 cases (69% decrease, 95% CI 18%–88%), (Figure 2). Five of the 6 cases had ST11 CC (3 were confirmed by PCR only, and ST11 CC war inferred for PorA P1.5.2). None of these 6 eligible case-patients had received MenACWY vaccine, and only 1 (a student from overseas who was not vaccinated) was in a university setting. On the basis of population coverage of 36.6% among persons who left school, early estimated vaccine effectiveness was 100% (95% CI −47% to 100%), but CIs were wide because of small numbers.

One case each of MenY and MenC disease were diagnosed in persons (both not vaccinated) who left school during 2015–16 compared with 3 of each in 2014–15. MenB cases increased from 10 in 2014–15 to 17 in 2015–16. Of the 17 case-patients in 2015–16, six (35%) had received MenACWY vaccine, consistent with national vaccine coverage.

## Conclusions

We found a major reduction (69%) in observed MenW cases compared with predicted MenW cases among the first cohort in England to be offered MenACWY conjugate vaccine after the first year of an emergency vaccination program for teenagers, even with a small number of cases. This decrease occurred despite national vaccine coverage of only 36.6% for this cohort. All case-patients who left school in 2015 and had confirmed MenW disease were not vaccinated; the only university case was in an overseas student who was not vaccinated. Higher vaccine coverage among university-affiliated general medical practices suggests that persons who left school and were enrolled in universities were more likely to be vaccinated than age-matched peers who did not seek higher education. Some universities have actively vaccinated new entrants and achieved high uptake rates (*11*).

Our initial data on vaccine effectiveness and effect on disease among persons who left school in 2015 are encouraging. However, continued surveillance is vital. Multicomponent MenB vaccine 4CMenB (Bexsero, Basel, Switzerland) was added to the UK national infant vaccination program for infants in September 2015 (*12*). Unlike conjugated polysaccharide meningococcal vaccines, 4CMenB is not capsule specific and has the potential to offer broader protection against all meningococcal strains. Antibodies from 4CMenB-vaccinated infants showed potent serum bactericidal antibody activity against the hypervirulent MenW ST11 strain (*13*), which is consistent with the observed decrease in MenW cases among infants.

Public Health England will continue to monitor effects of vaccination programs as more cohorts are vaccinated. Younger cohorts are receiving MenACWY conjugate vaccine through a school-based program; uptake rates are much higher (72%–84%) than for persons who left school and were vaccinated through general medical practices (*14*). Although overall MenW cases increased in 2015–16 compared with 2014–15, the proportionate increase in cases was lower than in previous years, when cases were nearly doubling every year. Whether this finding indicates early signs of an indirect effect is speculative, but 4 months into the 2016–17 academic year, total MenW case numbers are only 8% higher than at the same time in 2015–16.

By the autumn of 2017, MenACWY vaccine will have been offered to all targeted teenagers in the United Kingdom. Because this group also has the highest meningococcal carriage rates (*15*), we hope that preventing carriage through vaccination will reduce cases and deaths in unvaccinated cohorts across all age groups in the coming years.
